# Whether the infracardiac bursa protect right pleura during laparoscopic radical operation of Siewert type II adenocarcinoma of esophagogastric junction?

**DOI:** 10.1186/s12885-022-10024-5

**Published:** 2022-08-27

**Authors:** Zeyu Lin, Haiping Zeng, Wenjun Xiong, Jin Li, Yan Chen, Lijie Luo, Yansheng Zheng, Zhuoxuan Zhang, Wei Wang

**Affiliations:** 1 The Second School of Clinical Medicine, Guangzhou University of Chinese Medicine, Guangzhou, China; 2 Department of Gastrointestinal Surgery, Guangdong Provincial Hospital of Chinese Medicine, The Second Affiliated Hospital of Guangzhou University of Chinese Medicine, Dade Road No. 111, Guangzhou, 510120 China; 3 State Key Laboratory of Dampness Syndrome of Chinese Medicine, Guangdong Provincial Hospital of Chinese Medicine, Guangzhou, China

**Keywords:** Infracardiac bursa, Adenocarcinoma of the esophagogastric junction, Siewert type II, Five-step maneuver, Right pleura

## Abstract

**Background:**

Transthoracic single-port assisted laparoscopic five-step maneuver inferior mediastinal lymphadenectomy for Siewert type II adenocarcinoma of esophagogastric junction (AEG) has superiority in lower mediastinal lymph nodes dissection and digestive tract reconstruction. However, the right pleura was probably ruptured in this surgical technique. The aim of this study was to explore whether the infracardiac bursa (ICB) exposed could protect right pleura.

**Methods:**

We retrospectively collected and evaluated the clinical and pathological data of patients who underwent five-step maneuver of transthoracic single-port assisted laparoscopic lower mediastinal lymphadenectomy for Siewert II AEG at Guangdong Provincial Hospital of Chinese Medicine between May 2017 and February 2022.

**Results:**

A total of 49 patients were eligible, including 31 patients in ICB exposed group (group A) and 18 patients in ICB unexposed group (group B). There were no statistically significant differences in baseline characteristics between the two groups. 4 patients (12.9%) had right pleura rupture in group A, while 14 patients (77.8%) in group B, and the difference was statistically significant (*p* < 0.001). Compared with group B, the extubation time of endotracheal intubation (10.0 (6.0 ~ 12.0) vs. 13.0 (8.0 ~ 15.0) min, *p* = 0.003) and thoracic drainage tube stay (6.0 (5.0 ~ 7.0) vs. 8.0 (6.0 ~ 10.5) days, *p* = 0.041) were significantly shorted in the group A. The drainage volume of thorax (351.61 ± 125.00 vs. 418.61 ± 207.86 mL, *p* = 0.146) was non-significant less and the rate of complications (3.2% vs. 11.1%, *p* = 0.074) was non-significant lower in group A compared with group B. The postoperative hospital stay (9.0 (8.0,13.0) vs. 9.0 (8.0,12.0) days, *p* = 0.983) were similar in two groups. No serious adverse event occurred in any patient.

**Conclusions:**

The ICB exposed could protect the right pleura and may promote postoperative recovery, which may be used as an anatomical marker in inferior mediastinal lymphadenectomy.

## Introduction

The incidence of adenocarcinoma of esophagogastric junction (AEG) is increasing in different region [1–4]. Surgery is still the main curative treatment for the AEG [5]. Laparoscopic techniques have been used for AEG commonly [6–8]. It is reported that the rate of lower mediastinal lymph node metastasis in Siewert type II AEG is more than 10%, which is positively correlated with the length of esophageal invasion [9–11]. Therefore, inferior mediastinal lymph node dissection is necessary. However, the operative field is narrow and limited by surrounding organs, so it is of great difficulty to dissect inferior mediastinal lymph node and reconstruct digestive tract. Besides, the surgical approach for this tumor is still controversial. Our team put forward a new technique, named transthoracic single-port assisted laparoscopic lower mediastinal lymph node dissection for Siewert type II AEG [12], which could overcome the difficulties above, and the inferior mediastinal lymph nodes could be dissected completely. However, it is difficult to identify the right pleura during the operation, which may cause unintended pleural rupture. Right pleural rupture may affect the recovery of short-term respiratory function and increase the volume of pleural effusion after operation [13, 14]. With the accumulation of experience, we found that infracardiac bursa (ICB) [15] may play a role in protecting the right pleura. Therefore, the aim of this study was to explore whether the ICB exposed could protect right pleura during laparoscopic radical operation of Siewert type II adenocarcinoma of esophagogastric junction.

## Methods

### Patients

Patients who underwent five-step maneuver of transthoracic single-port assisted laparoscopic lower mediastinal lymphadenectomy for Siewert type II AEG at Guangdong Provincial Hospital of Chinese Medicine between May 2017 and February 2022 were included in the study. We collected the following clinical and pathological data: sex, age, body mass index (BMI), American Society of Anesthesiologists Score (ASA), T and N stage, time of lower mediastinal lymph nodes (LNs) dissection, number of lower mediastinal LNs dissection, right pleura rupture or unruptured, the ICB exposed or unexposed, drainage volume of thorax (based on the nursing record on the first day after surgery), extubation time of endotracheal intubation (defined as the time between the end of the surgery and the endotracheal intubation extubated), thoracic drainage tube stay, postoperative hospital stay. All surgical videos of the laparoscopic lower mediastinal lymphadenectomy were saved to verify the right pleura ruptured or unruptured and the ICB exposed or unexposed. Approval was granted by the ethics committee of Guangdong Provincial Hospital of Chinese Medicine (ZF2018-219). And all participants provided written informed consent.

### Surgical procedures

**Fig. 1:**
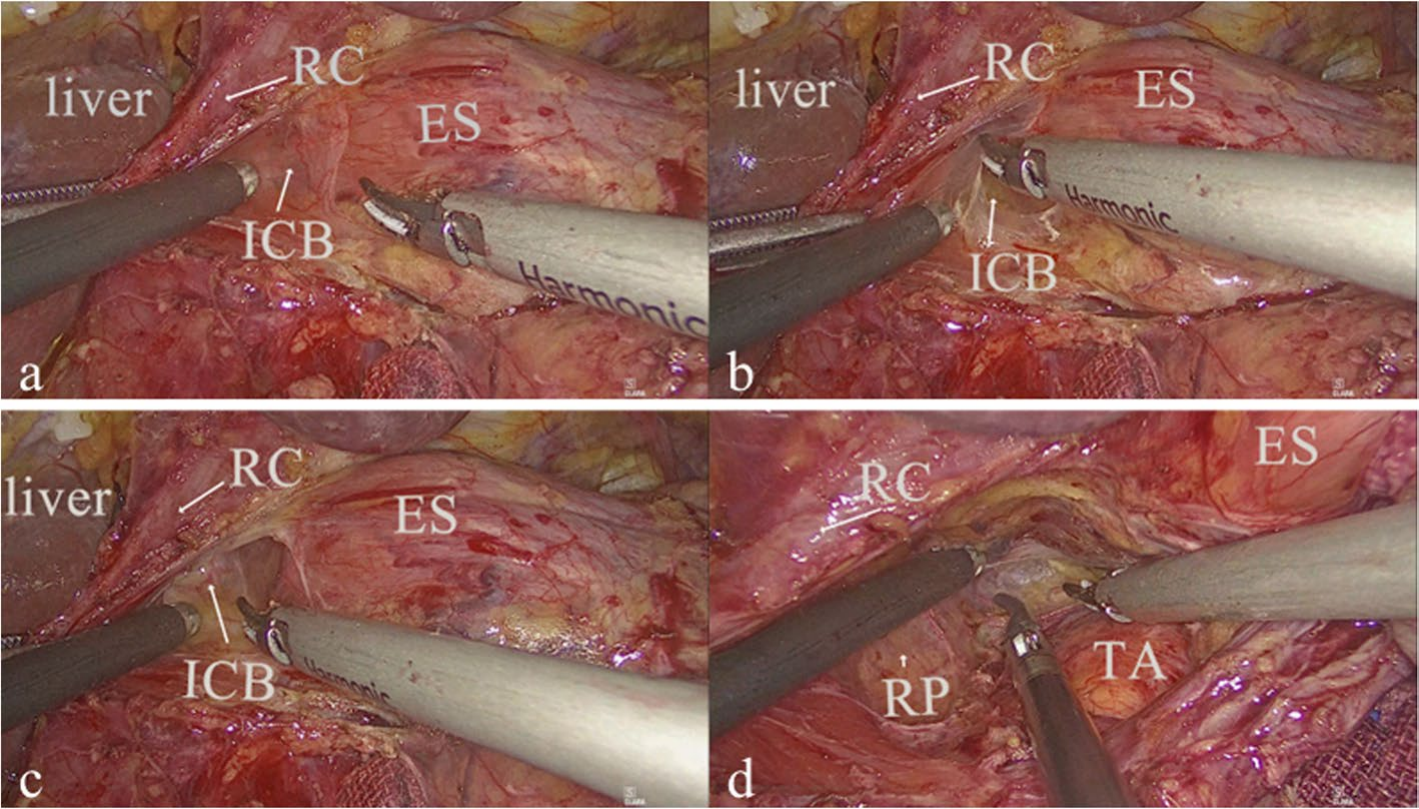
**a** before the ICB exposed. **b** the ICB was being exposed. **c** after the ICB exposed. **d** after the No.112pulR, and the No.112aoA dissected. RC, right diaphragmatic crus; ES, esophagus; ICB, infracardiac bursa; TA, thoracic aorta; RP, right pleura

Surgical procedures were performed as Five ⁃ step maneuver reported by our team previously during which the right pleura wasn’t routinely opened [16]. Key procedures were as follows: the perigastric lymph node dissection was performed according to D2 lymph node dissection based on the Japanese gastric cancer treatment guidelines [17]. And then, incising the phrenico-esophageal ligament surrounding the esophagus, the No.19, 20 lymph nodes were removed. After that, lower mediastinal lymph node dissection performed: firstly, the ICB exposed (The stomach was pulled down to the left. The ICB was cut or pushed to enter its closed space, the right boundary was protected to avoid the right pleura rupture. Figure 1), and posterior mediastinal lymph nodes (No.112pulR, No.112aoA) was dissected. Secondly, the diaphragm was incised and a 12 mm trocar was placed as another main operation hole, and the No.112pulL was dissected up to the left inferior pulmonary vein through it. Then, No.111 was dissected. After that, the posterior pericardium was denuded and the No.112pulR was completely removed. Finally, N0.110 was dissected and the esophagus was severed.

### Statistical analysis

Data were analyzed with SPSS software version 26 (IBM Corp., New York, USA). Continuous data was expressed as $\overline{x }$±s or Median (P_25_ ~ P_75_). Student’s t-test or Mann–Whitney U test was used to assess statistical significance. Categorical data was presented as numbers and ratio (%), and analyzed by using the Chi-squared or Fisher's exact tests. A *P*-value of < 0.05 was considered statistically significant.

## Results

A total of 49 patients were eligible for this study. Of these, 31 patients in ICB exposed group (group A) and 18 patients in ICB unexposed group (group B). Baseline demographics characteristics were well balanced between the two groups (Table 1).

**Table 1:** Baseline demographics characteristics between the two groups

Variables	ICB exposed group	ICB unexposed group	*P* value
	(group A, *n* = 31)	(group B, *n* = 18)	
Age(year)	63.74 ± 8.0	67.94 ± 11.30	0.135
Sex			0.443
Male	27(87.1)	14(77.8)	
Female	4(12.9)	4(22.2)	
BMI(Kg/m^2^)	22.60 ± 3.22	21.51 ± 3.64	0.286
ASA			0.217
II	23(74.2)	10(55.6)	
III	8(25.8)	8(44.6)	
T stage			0.069
2	0	2(11.1)	
3	18(58.1)	6(33.3)	
4	13(41.9)	10(55.6)	
N stage			0.131
0	3(9.7)	7(38.9)	
1	9(29.0)	3(16.7)	
2	10(32.3)	4(22.2)	
3	9(29.0)	4(22.2)	

The number of right pleura rupture in group A was less than that in group B, and the difference was statistically significant (*p* < 0.001). The median extubation time of endotracheal intubation and the median thoracic drainage tube stay was significantly shorter in the group A compared with the group B. The drainage volume of thorax in group A was less than that in group B, but the difference was not statistically significant. The time of lower mediastinal LNs dissection, the number of lower mediastinal LNs dissection, the median postoperative hospital stay, and the morbidities of postoperative complications were similar between these two groups. However, the mean postoperative hospital stay in the group A was shorter than that in group B, although this was not statistically significant. No serious adverse event occurred in any patient (Table 2).

**Table 2:** Perioperative outcomes between the group A and B

Variables	ICB exposed group	ICB unexposed group	*P* value
	(group A, *n* = 31)	(group B, *n* = 18)	
right pleura			0.0001
unruptured	27 (87.1)	4 (22.2)	
rupture	4 (12.9)	14 (77.8)	
time of lower mediastinal LNs dissection (min)	40.94 ± 9.69	38.28 ± 10.40	0.372
number of lower mediastinal LNs dissection	4.0(3.0 ~ 8.0)	5.0(3.0 ~ 6.0)	0.889
extubation time of endotracheal intubation (min)	10.0(6.0 ~ 12.0)	13.0(8.0 ~ 15.0)	0.003
thoracic drainage tube stay (days)	6.0(5.0 ~ 7.0)	8.0(6.0 ~ 10.5)	0.041
postoperative hospital stay(days)			
Median (P_25_ ~ P_75_)	9.0 (8.0 ~ 12.0)	9.0(8.0 ~ 13.0)	0.983
Mean ± SD	10.97 ± 3.87	14.67 ± 15.56	0.335
drainage volume of thorax(mL)	340(280 ~ 400)	430(243.8 ~ 550)	0.295
Postoperative complications			0.074
pancreatic fistula	1	0	
pleural effusion	0	2	
anastomotic leakage	0	1	

Then, the entire cohort was divided into two groups, according to whether right pleura ruptured or unruptured (rupture group and unruptured group), to evaluate the influence upon postoperative outcomes (Table 3). The median extubation time of endotracheal intubation and the median thoracic drainage tube stay in the unruptured were significantly shorter than those in the rupture group. Compared with rupture group, the drainage volume of thorax was less and the median postoperative hospital stay was shorter in unruptured group, but these trends did not achieve statistical significance.

**Table 3:** Perioperative outcomes between unruptured and rupture group

Variables	unruptured group (*n* = 31)	rupture group (*n* = 18)	*P* value
extubation time of endotracheal intubation (min)	6.0 (8.0 ~ 10.0)	13.0 (9.5 ~ 15.5)	0.004
Thoracic drainage tube stay (days)	6.0 (5.0 ~ 7.0)	8.0 (6.0 ~ 10.5)	0.028
drainage volume of thorax (mL)	340 (275 ~ 380)	450 (272.5 ~ 512.5)	0.198
postoperative hospital stay (days)	10.0 (9.0 ~ 12.0)	10.5 (8.0 ~ 12.3)	0.842

## Discussion

In this study, compared with the ICB unexposed group, the number of right pleural rupture in the ICB exposed group were greatly decreased, and the extubation time of endotracheal intubation and the thoracic drainage tube stay were shorter. Moreover, the drainage volume of thorax and rate of complications tended to be less. The postoperative hospital stay was similar in the two groups.

Surgery is still the main means to improve the survival rate of advanced AEG. The Siewert classification is widely used for determining which surgical approach is selected. Siewert types I and III are treated as esophageal cancer and gastric cancer, respectively. However, there was no consensus about surgical approach has been reached for the Siewert type II AEG [18]. Besides, the special anatomical position of Siewert type II AEG resulted in many challenges during surgery. Our team proposed a novel technique, transthoracic single-port assisted laparoscopic five-step maneuver lower mediastinal lymphadenectomy, which could effectively solve the technical difficulties in terms of inferior mediastinal lymph node dissection and digestive tract reconstruction.

With the application of this technique, we found that the right pleura may rupture when the right boundary (right pleura) was dissected in inferior mediastinal lymph node dissection. The pleural rupture rate of laparotomy (transabdominal esophageal hiatus) reported in previous studies was 36.4%, and that in laparoscopic esophageal hiatus approach was 18.4% to 30% [13, 19]. In our study, the rupture rate of the right pleura in the ICB unexposed group was 77.8% (14/18). However, we gradually found that the exposure of the ICB may make up for this deficiency. The ICB is a widely-known derivative separated from the omental bursa in embryology. It was demonstrated that the right pneumato-enteric recess originated from superior part of the omental bursa was incised by the developing diaphragm, which separated a closed space called ICB [20]. Tatsuro et al. indicated that the ICB was the structure universally remaining in almost all adults, and located at the right alongside the esophagus and the cranial side of the diaphragmatic crus [15]. Because of this unique anatomical location, the ICB may protect the right pleura during the surgery. The right pleural rupture rate in the exposed ICB group is 12.9% (4/31) in our study is consistent with previous inference, revealing that ICB exposed can effectively reduce the rupture in right pleura. It is reported that pleural rupture has an adverse effect on the recovery of respiratory function, which may prolong the recovery time of early postoperative respiratory function [13]. In our study, extubation time of endotracheal intubation was longer in the ICB unexposed group. Therefore, the short-term recovery of respiratory function may be affected by the destruction of the right pleura, and the ICB exposed may benefit to the recovery of respiratory function.

Under the guidance of the concept of Enhanced Recovery After Surgery, reducing or not placing drainage tubes can accelerate the recovery of patients [21, 22]. At the same time, the reduction of drainage tube can reduce the discomfort of patients' out-of-bed activity, which may increase their willingness to out-of-bed. Early mobilization is beneficial to patients' rehabilitation and prevention of complications such as pneumonia, thromboembolism, muscle wasting [23]. We found that the thoracic drainage tube was extubated earlier in the exposed ICB group Moreover, the mean postoperative hospital stay was shorter in the exposed ICB group, although this difference didn’t achieve statistical significance. It reaches a similar result when the entire cohort was divided into another two groups (rupture group and unruptured group), which suggest that the patient with the right pleura protected may recovery more quickly.

Since the low rate of postoperative complications, the new surgical approach that transthoracic single-port assisted laparoscopic five-step maneuver inferior mediastinal lymphadenectomy is safe for treating Siewert type II adenocarcinoma of esophagogastric junction. Moreover, it would not affect lower mediastinal lymph nodes dissection for the similar time of dissection and number of lymph nodes. It is report that the rate of operative complications was 33.4% in an esophagectomy via a transthoracic approach and 27.6% in a gastrectomy via a transhiatal approach [24]. However, in our study, the rate of operative complications was only 3.1% in exposed group and 11.1% in unexposed group. It showed that the postoperative complications in our new surgical approach may be controlled within a reasonable range. Japanese JCOG9502 study reported that the median number of mediastinal lymph nodes in the TH group was 2 [25]. However, the median number of lower mediastinal LNs in ICB exposed and ICB unexposed group were 4.5 and 5.0, respectively. And these results showed that there was a superiority of lower mediastinal lymph nodes dissection in our surgical approach.

There are several shortcomings in this study: First, this is a retrospective study, and the sample size is small. Second, without Computed Tomography or B-ultrasound examination to assess the patient's pleural effusion after surgery. Third, though the average postoperative hospital stay was shorter in exposed ICB group, the median postoperative hospital stay was similar. Last, the cases collected in the study belong to a feasibility and efficacy study of this new surgical approach, transthoracic single-port assisted laparoscopic Siewert type II AEG radical operation, which may have an impact on the length of hospital stay. Despite these limitations, our results indicated that the ICB exposed could protect the right pleura.

## Conclusion

The ICB exposed could protect the right pleura and may promote postoperative recovery, which may be used as an anatomical marker in inferior mediastinal lymph nodes dissection. Further research is warranted.

## Data Availability

The datasets generated and/or analysed during the current study are not publicly available but are available from the corresponding author on reasonable request.

## Abbreviations

AEG: Adenocarcinoma of esophagogastric junction
ICB: Infracardiac bursa
LNs: Lymph nodes
